# Supplementary material for: Selection and Validation of Reference Genes for RT-qPCR Analysis of Gene Expression in Nicotiana benthamiana upon Single Infections by 11 Positive-Sense Single-Stranded RNA Viruses from Four Genera
Source: Plants (Basel). 2023 Feb 14;12(4):857. doi: 10.3390/plants12040857 (PMC9964245; doi:10.3390/plants12040857)
Supplement: Supplementary file 1 [file plants-12-00857-s001.zip › plants-2197019-supplementary.pdf]

Table S1 Information of the 28 selected potential RT-qPCR reference genes and primer sequences

Genes	Full Names	Forward/reverse primers (5'-3')	References
NbeIF4A	ATP-dependent RNA helicase eIF4A	GCTTTGGTCTTGGCACCTACTC/AAGGATGCGTTGATCCTCAC	37
NbLip	Lipoyl synthase	GAGAAGTACACGCAGATTAAGA/CATTGCAGAAACAACACTCTC	38
NbL23	60S ribosomal protein	AAGGATGCCGTGAAGAAGATGT/GCATCGTAGTCAGGAGTCAACC	29
NbKLC	Kinesin light chain	GATGACTCTCTCTGCGATTCTG/TGCACTTGAGGACAAGGAATTA	38
NbEF1α	Elongation factor 1α	AGCTTTACCTCCCAAGTCATC/AGAACGCCTGTCAATCTTGG	29
NbPGK	phosphoglycerate kinase	CAGACATCGTTCCGTTCATC/CCCTTCAGATCTGCTTCTTTC	38
NbTspan	Tetraspanin-20	TGTCGCTGTTCTGCTTTC/CACGCCATTCTCTACACTTC	38
NbRdR6	Putative RNA-dependent RNA polymerase SDE1	TTCAGGAATGTCTTCGAGCG/AGTGATCTAGCAACCCAATGAG	29
NbF-BOX	F-box protein	GGCACTCACAAACGTCTATTTC/CTGGGAGGCATCCTGCTTAT	29
NbUBC	ubiquitin-conjugating enzyme 3	AAGCCGGCGACGAACATAAG/CGGAATTGTTGACCTACGAG	This study
NbACT	Actin	AAAGACCAGCTCATCCGTGGAGAA/TGTGGTTTCATGAATGCCAGCAGC	39
NbAGO2	Putative Argonaute-2 protein	CATTTGAACCTCCTTTCTATCGAC/CATACCTCTAGAAGTGAGGATCAC	29
NbGAPDH	Glyceraldehyde 3-phosphate dehydrogenase	AGCTCAAGGGAATTCTCGATG/AACCTTAACCATGTCATCTCCC	29
NbACT3	Actin-3	CCAGAGAGGAAATACAGTG/CAATAGACGGACCAGATTCG	40
NbEF1	Elongation factor 1-α 3	GACAAGCGTGTTATTGAGAGG/CACAGTGCAGTAGTACTTAGTG	41

NbPP2a	Protein phosphatase 2a	GACCCTGATGTTGATGTTCGCT/GAGGGATTTGAAGAGAGATTTC	29
NbPPR	Pentatricopeptide repeat containing protein	ATGAGGGTCCATTTGAGTGAC/AGGCTGATGTTGGAATCTGG	29
NbTIP41	TIP41-like protein	ACGAGGATGAATTGGCCGATAA/CCAGAAACGCAGCAATAGGAAC	29
NbTUB	β-Tubulin	CAAGATGCTACTGCAGACGAG/CTGGAAGTTGTGGTTTTGGC	29
NbUK	Uridylate kinase	CTAGGAGTATATTGGAAGAGCG/AAAGATACATCGCCTTGCTGAA	29
NbAPR	Adenine phosphoribosyltransferase like	CATCAGTGTCGTTGCAGGTATT/GCAACTTCTTGGGTTTCCTCAT	29
NbCENPO	Centromere protein O	TACCACTGCGTGCTTGAAA/GATCTCCGACATGGCCAATAA	38
NbUbe35	ubiquitin-conjugating enzyme 35	CTTCAGATTCGCACCGTTCT/CCAATGCTTCGCAATGTTCTC	38
NbNQO	NAD(P)H dehydrogenase (quinone)	AAGGCGGTGGTCAAGAAA/CAAACATACCAGCACCGAATG	38
NbNAC042	NAC domain-containing protein 68	CAAACAGGGTGACAGGTTCT/ATGACTTCTTCAGCCCAATACA	38
NbErpA	Iron-sulfur cluster insertion protein ErpA 2	GCTTGGACCACTGGATATAAA/CATTCCCAGTTACACCATTAGA	38
NbGBP	GTP binding protein	GGAACTGGATTCGCAACATAGA/GACCCTTGGAAGTTGGCACAGC	29
NbP5βR	progesterone 5-beta-reductase 1	CCTCTTTCTCTTCACTCACTCTC/TTAGAGCTACGCTTTGGTACTT	38

Notes: Genes labeled in black letters had a single-peaked melting curve in RT-qPCR and showed only one band after ethidium bromide staining. Red-lettered genes had double peaks or a red curve in the melting curve. Blue-lettered genes had a sequence that did not match the expected target gene sequences, as confirmed by Sanger sequencing of at least three clones of RT-qPCR products cloned into pGEM®-T vector.

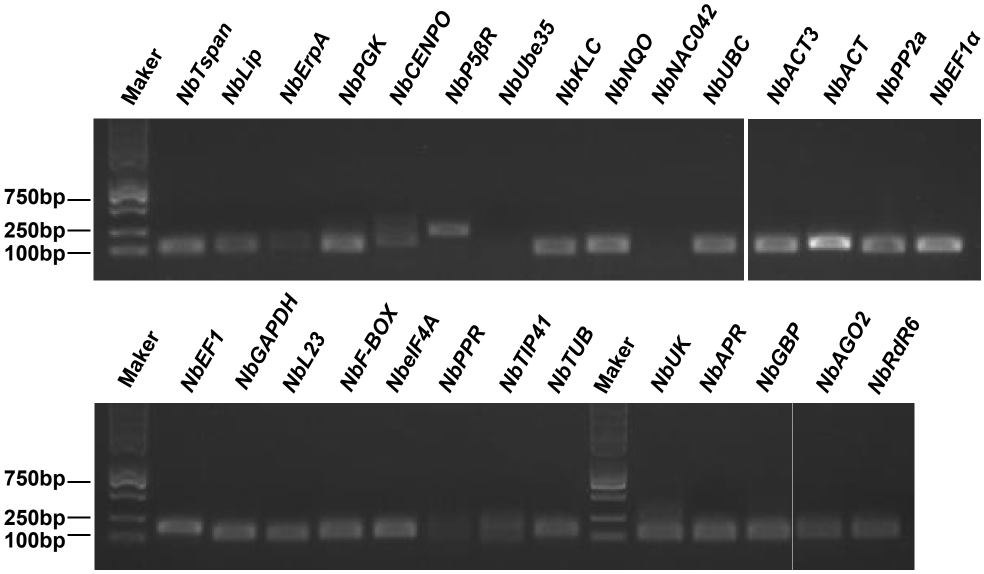

Figure S1 Analysis of RT-qPCR product of 28 genes through 1% agarose gel electrophoresis and ethidium bromide staining

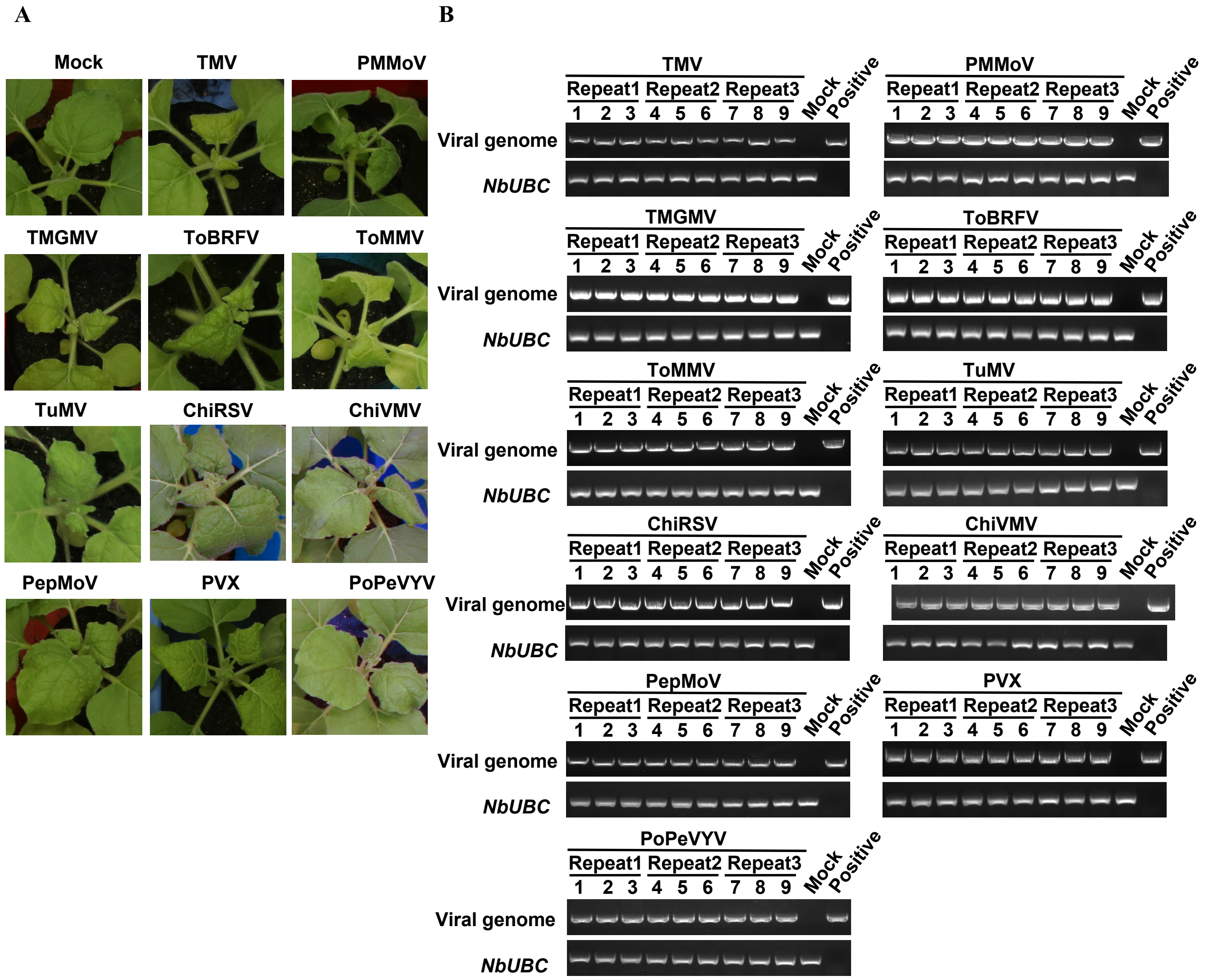

Figure S2 Symptoms induced by infections of 11 viruses from four genera (A) and RT-PCR confirmation of viral infection (B)

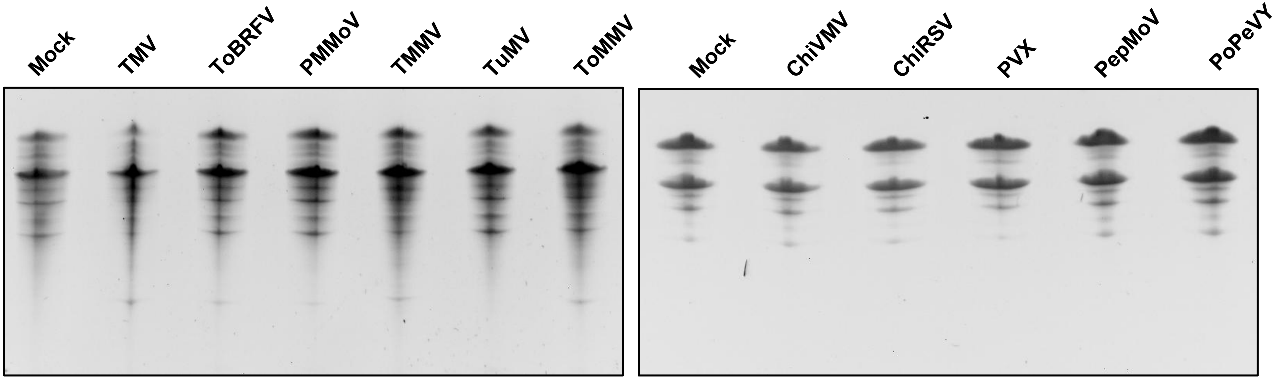

Figure S3 Analysis of RNA integrity through 5% urea-PAGE gel electrophoresis and ethidium bromide staining

Table S2 Information of expression vectors of the 11 viruses used in this study

Viruses	Genome length	Backbone vector	Promoter
TMV	6396bp	pCB301	35S
PMMoV	6356bp	pCB301	35S
TMGMV	6356bp	pCB301	35S
ToBRFV	6391bp	pCB301	35S
ToMMV	6398bp	pCB301	35S
TuMV	9835bp	pCB301	35S
ChiRSV	9652bp	pCB301	35S
ChiVMV	9725bp	pCB301	35S
PepMoV	9640bp	pCB301	35S
PVX	6652bp	pGreen	35S
PoPeVYV	6015bp	pCB301	35S

Table S3 Sequences of primers used in RT-PCR to confirm viral infection

Viruses	Primers	Sequences	PCR product length (bp)
TMV	TMV-F	AAGGATTTTGGAGGAATGAG	587
	TMV-R	TTATGCATCTTGACTACCTC	
PMMoV	PMMoV-F	GCCGGAATAAAAACGTGTTTG	1637
	PMMoV-R	GGCAGTTGTAGGATTTTGCG	
TMGMV	TMGMV-F	CAAATGCATTGGGTAACCAG	380
	TMGMV-R	TGTGGTCCAGACAAGTCCAC	
ToBRFV	ToBRFV-F	ATGTCTTACACAATCGCAACTC	459
	ToBRFV-R	CCATTGTAAACCGGATGCAC	
ToMMV	ToMMV-F	CTAAGTCAGAAAGGTTGCTG	1265
	ToMMV-R	CACTTCCTTTAATTTCGTCC	
TuMV	TuMV-F	CTTGATGCAGGTTTGACAGA	618
	TuMV-R	ATTGCGCTGAAGACCATATC	
ChiRSV	ChiRSV-F	CACAAGCCATAGATGCTGG	567
	ChiRSV-R	GCTGCAAACCATACCTTGGC	
ChiVMV	ChiVMV-F	GGAGAGAGCGTTGATGCTG	581
	ChiVMV-R	TTGCGCTTCTCAATGTACGC	
PepMoV	PepMoV-F	AGCAGCTCAAGATCAGAAACAC	822
	PepMoV-R	TCATATATTCCTGACCCCAAGCA	
PVX	PVX-F	CAATCACAGTGTTGGCTTGC	556
	PVX-R	CCGTTGGAATAGGGACCTGT	
PoPeVYV	PoPeVYV-F	AAGACGACGAAATGGAGGC	487
	PoPeVYV-R	CTATTTGGGGTTGTGCAGTTG	
NbUBC	UBC-F	TTTCGGTCCTGATGATACTCCC	224
	UBC-R	CACAGAGCAAAGACTGGATTGA	
